# CRL4–DCAF1 ubiquitin E3 ligase directs protein phosphatase 2A degradation to control oocyte meiotic maturation

**DOI:** 10.1038/ncomms9017

**Published:** 2015-08-18

**Authors:** Chao Yu, Shu-Yan Ji, Qian-Qian Sha, Qing-Yuan Sun, Heng-Yu Fan

**Affiliations:** 1 Life Sciences Institute and Innovation Center for Cell Signaling Network, Zhejiang University, Hangzhou 310058, China; 2 State Key Laboratory of Reproductive Biology, Institute of Zoology, Chinese Academy of Sciences, Beijing 100101, China

## Abstract

Oocyte meiosis is a specialized cell cycle that gives rise to fertilizable haploid gametes and is precisely controlled in various dimensions. We recently found that E3 ubiquitin ligase CRL4 is required for female fertility by regulating DNA hydroxymethylation to maintain oocyte survival and to promote zygotic genome reprogramming. However, not all phenotypes of CRL4-deleted oocytes could be explained by this mechanism. Here we show that CRL4 controls oocyte meiotic maturation by proteasomal degradation of protein phosphatase 2A scaffold subunit, PP2A-A. Oocyte-specific deletion of DDB1 or DCAF1 (also called VPRBP) results in delayed meiotic resumption and failure to complete meiosis I along with PP2A-A accumulation. DCAF1 directly binds to and results in the poly-ubiquitination of PP2A-A. Moreover, combined deletion of *Ppp2r1a* rescues the meiotic defects caused by DDB1/DCAF1 deficiency. These results provide *in vivo* evidence that CRL4-directed PP2A-A degradation is physiologically essential for regulating oocyte meiosis and female fertility.

## Abstract (web summary)

The E3 ubiquitin ligase CRL4 regulates oocyte survival through hydroxymethylation of genomic DNA. Here Yu *et al*. show that CRL4 is also required for oocytes to complete meiosis I by mediating the poly-ubiquitination and proteasomal degradation of the cell cycle regulator protein phosphatase 2A-A subunit.

## Graphical abstract

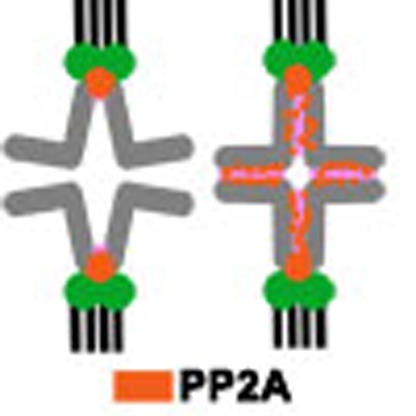

To produce fertilizable eggs with the appropriate number of chromosome sets, oocyte meiotic progression is precisely regulated^1^. In ovaries of adult female mammals, oocytes are arrested at the diplotene stage of prophase I, which is also called germinal vesicle (GV) stage^2^. A luteinizing hormone surge at oestrus triggers oocyte meiotic resumption in preovulatory follicles, followed by chromosome alignment and spindle organization at prometaphase I (Pro-MI)^3^,^4^. Accurate homologous chromosome separation is achieved by cohesion removal after all chromosome pairs are aligned along the spindle equatorial plane^5^,^6^. Subsequently, oocytes extrude a first polar body (PB1) and are arrested at metaphase II (MII) to await fertilization. Dysregulated meiotic progression results in aneuploidy and embryonic development abnormality, which contributes to early abortion and female infertility^7^.

Protein phosphatase 2A (PP2A) is a known cell cycle regulator in both somatic cells and oocytes. PP2A consists of a scaffold A subunit, a catalytic C subunit and a regulatory B subunit^8^. Previous studies showed that increased PP2A activity inhibited oocyte GV breakdown (GVBD) by counteracting CDK1 activation^9^. In contrast, genetically deleting *Ppp2r1a* in oocytes, a gene encoding for the major isoform of the PP2A-A subunit, facilitated GVBD, which suggested that PP2A functioned as an inhibitor of meiotic resumption^10^. In addition, PP2A is required to prevent precocious separation of sister chromatids during oocyte meiosis I^10^,^11^. During meiotic division I, sister chromatids are attached to one another by cohesin complexes when homologous chromosomes form tetrads. During the MI-to-AI transition, cohesins on chromosome arms are phosphorylated and then cleaved by separase, while centromeric cohesins are protected by a PP2A-shugoshin complex through dephosphorylation^5^,^6^,^11^,^12^. Although PP2A controls meiotic resumption and ensures accurate chromosome separation, little is known regarding how PP2A protein stability is regulated during cell cycles.

Protein ubiquitin (Ub) E3 ligases, in particular, play key roles during both meiotic and mitotic cell cycle progression by triggering specific proteins degradation. For example, an anaphase promoting complex (APC) initiates the metaphase–anaphase transition by inducing the degradation of cyclin B and securin^13^. Cullin-ring ligase-1 (CRL1) promotes early mitotic inhibitor 1 degradation and facilitates oocyte meiotic resumption and progression to MI^14^. However, it remains unclear whether PP2A is also regulated by the ubiquitination–proteasome pathway as part of the biochemical network that controls the cell cycle.

CRL4 Ub E3 ligase is evolutionally conserved from yeasts to humans. Its core components include cullin 4A or B as a scaffold, damaged DNA-binding protein 1 (DDB1) as a linker, and the ring finger protein ROC1/2 (refs 15, 16). By selectively forming protein complexes with its more than 90 substrate adaptors known as DDB1-CUL4 associated factors (DCAFs), CRL4 regulates a wide range of cellular processes^17^,^18^. We previously generated oocyte-specific *Ddb1* and *Dcaf1* knockout mice and found that CRL4 in complex with one of its substrate adaptors, DCAF1, also known as VPRBP, regulated the hydroxymethylation of genomic DNA to maintain oocyte survival and to promote zygotic genome reprogramming after fertilization^19^,^20^. Interestingly, although these knockout mice could ovulate in response to exogenous gonadotropin when they were young, most of their ovulated oocytes had abnormal morphologies, as characterized by the presence of GVs and the absence of PB1s. This suggested that, in addition to maintaining the survival of dormant oocytes, CRL4^DCAF1^ was a crucial regulator of meiotic maturation in fully grown oocytes.

In this study we investigate the physiological functions and biochemical mechanisms of CRL4^DCAF1^ in regulating mammalian oocyte meiotic maturation. DDB1 or DCAF1 deletion causes delayed meiotic resumption and failure to complete meiosis I in fully grown oocytes. The PP2A-A subunit is accumulated in DDB1- or DCAF1-deleted oocytes, which inhibits cohesin removal and homologous chromosome separation during meiosis I. Biochemistry assays shows that CRL4^DCAF1^ binds to PP2A-A and targets it for poly-ubiquitination and proteasome degradation. Our results demonstrate for the first time that CRL4^DCAF1^ Ub E3 ligase is essential for promoting meiotic cell cycle progression by targeting PP2A for degradation in mouse oocytes.

## Results

### CRL4^DCAF1^ complexes are essential for oocyte meiosis

**Figure 1:**
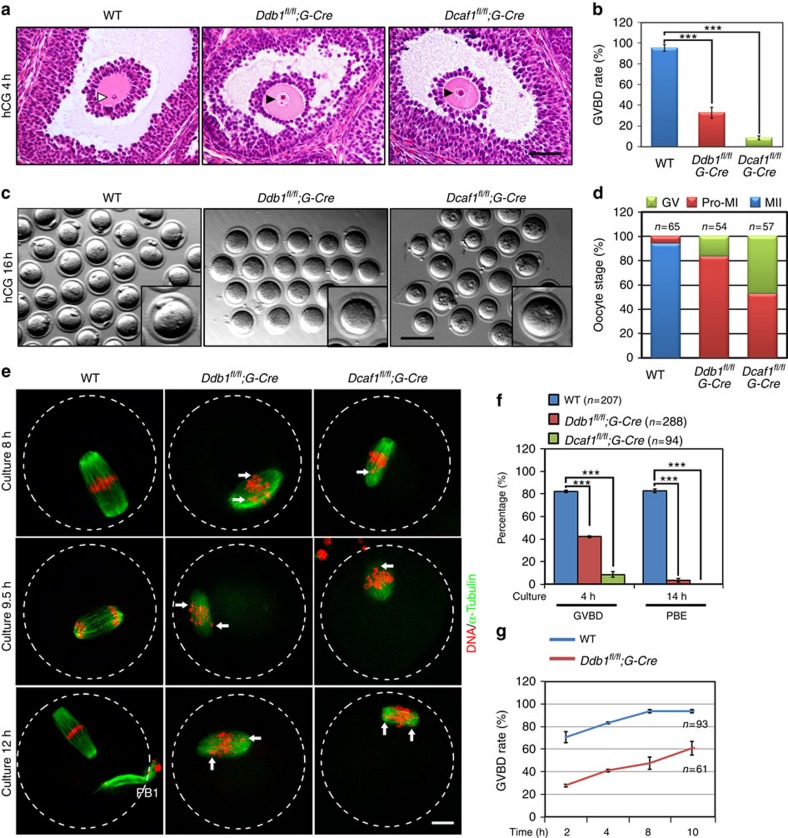
DDB1 or DCAF1 deletion in mouse oocytes causes meiotic maturation defects. (**a**) H and E stained images showing condensed chromosomes (indicated by white arrowheads) in preovulatory follicle-enclosed oocytes of WT mice (postnatal day (PD) 23–28) at 4 h post-hCG injection. However, in preovulatory follicles of *Ddb1*^*oo*−/−^ or *Dcaf1*^*oo*−/−^ mice, most oocytes still contained an intact GV (indicated by black arrowheads). Scale bar, 50 μm. (**b**) GVBD rates of preovulatory follicle-enclosed oocytes in mice with the indicated genotypes (PD23-28). *G-Cre* is an abbreviation for *Gdf9-Cre*. ***P<0.001, Student's *t*-test. (**c**) Images of ovulated oocytes from WT, *Ddb1*^*fl/fl*^*;Gdf9-Cre* and *Dcaf1*^*fl/fl*^*;Gdf9-Cre* females at 16 h after hCG injection. Scale bar, 100 μm. (**d**) Proportions of ovulated oocytes at GV, Pro-MI and MII stages shown in (**c**). Total numbers (*n*) of observed oocytes are indicated. (**e**) Microscopic imaging of WT (*n*=35), *Ddb1*^*oo*−/−^ (*n*=28) and *Dcaf1*^*oo*−/−^ (*n*=20) oocytes at the indicated time points. Dashed lines indicate oocyte outlines and white arrows indicate lagging chromosomes. Scale bar, 10 μm. (**f**) GVBD and PBE rates of cultured oocytes that were collected at the GV stage from WT, *Ddb1*^*oo*−/−^ and*Dcaf1*^*oo*−/−^ mice. Total numbers of oocytes used (*n*) are indicated. ***P<0.001, Student's *t*-test. (**g**) GVBD rates of cultured WT and *Ddb1*^*oo*−/−^ oocytes. Error bars indicate s.e.m.

To investigate the functions of CRL4^DCAF1^ complexes during oocyte meiotic maturation, we selectively deleted *Ddb1* or *Dcaf1* in oocytes by using previously reported conditional knockout mouse models, *Ddb1*^*fl/fl*^*;Gdf9-Cre* and *Dcaf1*^*fl/fl*^*;Gdf9-Cre* (hereafter referred to as *Ddb1*^*oo*−/−^ and *Dcaf1*^*oo*−/−^)^19^. Immunohistochemistry results confirmed that DDB1 and DCAF1 were specifically deleted in oocytes from *Ddb1*^*oo*−/−^ and *Dcaf1*^*oo*−/−^ mice, respectively (Supplementary Fig. 1). We then investigated the meiotic maturation of DDB1- and DCAF1-deficient oocytes. In wild-type (WT) females, 95% of the oocytes within preovulatory follicles resumed meiosis and underwent GVBD at 4 h after human chorionic gonadotropin (hCG) administration (Fig. 1a, white arrowheads). However, in the ovaries of *Ddb1*^*oo*−/−^ and *Dcaf1*^*oo*−/−^ mice, most oocytes within preovulatory follicles had intact GVs (Fig. 1a, black arrowheads) at 4 h post-hCG injection. Only 32.9 and 8.7% of these oocytes underwent GVBD (Fig. 1b). We superovulated WT, *Ddb1*^*oo*−/−^ and *Dcaf1*^*oo*−/−^ females and collected oocytes at 16 h post-hCG injection. GVBD occurred in 83% of *Ddb1*^*oo*−/−^ oocytes, but none of these oocytes extruded PB1s. More severe defects were observed for *Dcaf1*^*oo*−/−^ oocytes, as only 52.6% of the ovulated oocytes underwent GVBD and none released a PB1 (Fig. 1c,d). Cultured *Ddb1*^*oo*−/−^ and *Dcaf1*^*oo*−/−^ oocytes also had significantly low GVBD rate and extruded no PB1s *in vitro* (Fig. 1e–g and Supplementary Fig. 2a). For the reason that DDB1 protein is more stable than DCAF1, *Dcaf1*^*oo*−/−^ oocytes exhibited stronger phenotypes than *Ddb1*^*oo*−/−^ oocytes^19^.

In addition, *Ddb1*^*oo*−/−^ and *Dcaf1*^*oo*−/−^ oocytes that resumed meiosis had abnormal spindles and misaligned chromosomes (Supplementary Fig. 2b, arrows). When superovulated *Ddb1*^*oo*−/−^ females were mated with fertile adult males, 2-cell embryos were not found in their oviducts at 44 h post-hCG injection (Supplementary Fig 2c,d).

We then monitored the meiotic maturation of WT and DDB1-deleted oocytes *in vitro* using live imaging. mRNAs encoding for green fluorescent protein (GFP)-tubulin and RFP-H2B were co-injected into oocytes to demonstrate spindle assembly and chromosome organization. Compared with WT oocytes, GVBD was delayed in DDB1-deleted oocytes. Even after GVBD, these oocytes failed to emit a PB1 and did not complete meiosis I. Although spindles were formed in these oocytes, condensed chromosomes did not align at the equatorial plane (Supplementary Fig. 3).

In DDB1- or DCAF1-deleted oocytes that resumed meiosis within 4 h, lagged chromosomes were frequently observed, as in matured oocytes *in vivo* (Fig. 1e and Supplementary Fig. 2a). Homologous chromosome separation and PB1 emission (PBE) were not observed in these oocytes (Fig. 1e).

Moreover, when WT oocytes were cultured with MLN4924 (0.1–0.5 μM)^21^,^22^, a specific inhibitor of cullin neddylation that inhibits CRL family E3 ligase activity, homologous chromosome alignment and separation and PB1 extrusion were all disrupted (Supplementary Fig. 4). These phenotypes were similar to those observed with *Ddb1*^*oo*−/−^ and *Dcaf1*^*oo*−/−^ oocytes.

### Loss of CRL4 inhibits homologous chromosome separation

**Figure 2:**
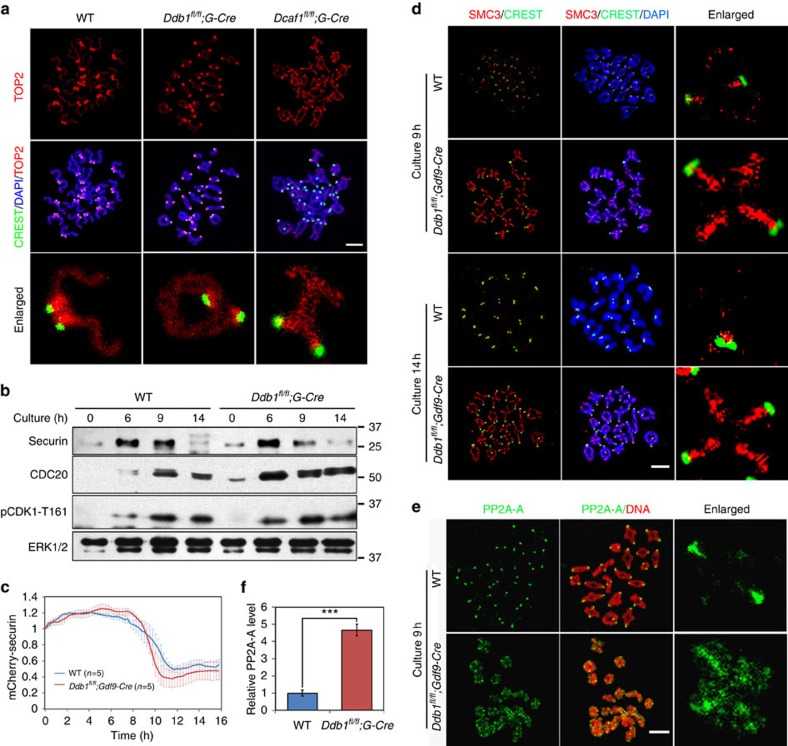
Homologous chromosome separation and cell cycle progression defects in DDB1 and DCAF1 deficient oocytes. (**a**) In ovulated oocytes of *Ddb1*^*oo*−/−^ or *Dcaf1*^*oo*−/−^mice, homologous chromosomes failed to separate, whereas typical bivalent chromosomes with paired sister chromatids were in ovulated WT oocytes. Chromosome configurations are shown by immunofluorescent staining for TOP2 (red) and CREST (green). DNA was labelled by DAPI (blue). Scale bar, 5 μm. (**b**) Western blotting results showing activation of APC/C complexes (accumulation of CDC20 and degradation of securin) in *Ddb1*^*oo*−/−^ oocytes. WT and *Ddb1*^*oo*−/−^ oocytes were cultured for the indicated times. Phosphorylation of CDK1-T161 was used as markers of meiotic cell cycle progression. Total proteins from 100 oocytes were loaded in each lane. (**c**) Data represent the mean and s.d. of mCherry-securin fluorescence intensity levels in WT (blue) and DDB1-deleted (red) oocytes at each time point. Time after release from milrinone is indicated. Values from individual oocytes were normalized relative to that at 0 h. (**d**) Representative images of SMC3 staining (red) showing cohesin removal from chromosomes of WT and *Ddb1*^*oo*−/−^ oocytes at indicated time points. Centromeres were labelled by CREST staining (green). DNA was labelled by DAPI (blue). Scale bar, 5 μm. (**e**) Representative images showing PP2A-A localization (green) on chromosomes of WT and *Ddb1*^*oo*−/−^ oocytes. Cultured oocytes were subjected to chromosome spreading at 9 h after culture. DNA was labelled by propidium iodide (red). Scale bar, 5 μm. (**f**) Quantification of PP2A-A level on the chromosomes of WT (*n*=8) and *Ddb1*^*oo*−/−^ (*n*=6) oocytes. Error bars indicate s.e.m. ***P<0.001, Student's *t*-test.

Chromosome spread analyses showed that the separation of homologous chromosomes was blocked in *Ddb1*^*oo*−/−^ and *Dcaf1*^*oo*−/−^ oocytes (Fig. 2a). At meiosis I, after homologous chromosome alignment and inactivation of the spindle assembly checkpoint (SAC), APC is activated by forming an APC^CDC20^ complex and targets securin and cyclin B for degradation, which activates separase and promotes anaphase^23^. Thus, the defects in homologous chromosome separation might have been caused by the inactivation or delayed activation of APC^CDC20^. To investigate this, we cultured WT and *Ddb1*^*oo*−/−^ oocytes for the indicated times and lysed them for western blot analysis. In WT oocytes, CDC20 accumulated at 9 h of culture and securin degraded thereafter, which indicated APC^CDC20^ activation (Fig. 2b). CDK1-T161 phosphorylation was not significantly affected by DDB1 deletion (Fig. 2b). Moreover, we microinjected mRNA encoding mCherry-securin into WT and *Ddb1*^*oo*−/−^ oocytes, and monitored the dynamics of mCherry fluorescent levels by live imaging. As shown in Fig. 2c, securin was normally degraded in DDB1-deleted oocytes. Thus, APC^CDC20^ was activated in DDB1-deleted oocytes, and targeted securin for degradation. The corresponding movies were shown in Supplementary Movie 1 (WT oocytes) and Supplementary Movie 2 (*Ddb1*^*oo*−/−^ oocytes), respectively. Consistent with the activation of APC^CDC20^, MAD2, a core component of SAC which concentrates to the centromeres when SAC is active, was not detected in the chromosomes of DDB1-deleted oocytes (Supplementary Fig. 5). These results suggested that APC and separase were activated but failed to separate homologous chromosomes.

During the MI-to-AI transition, cohesin on homologous chromosome arms that holds them together is phosphorylated and cleaved by separase^24^. In WT oocytes, structural maintenance of chromosomes 3 (SMC3), a component of the cohesin complex, was localized to the chromosome axis at early MI, but was removed from chromosome arms and restrictively localized to centromeres at the late MI and MII stages (Fig. 2d). However, in *Ddb1*^*oo*−/−^ oocytes, SMC3 was retained on the chromosome axis even when the WT oocytes have extruded PB1 (Fig. 2d). Centromeric cohesins that hold sister chromosomes together are protected by PP2A-shugoshin complexes^25^,^26^. In WT oocytes, PP2A was restricted to centromeres during the MI–AI transition (Fig. 2e). However, increased PP2A-A levels and ectopic PP2A localization on chromosome arms were observed in DDB1-deleted oocytes (Fig. 2e,f).

**Figure 3:**
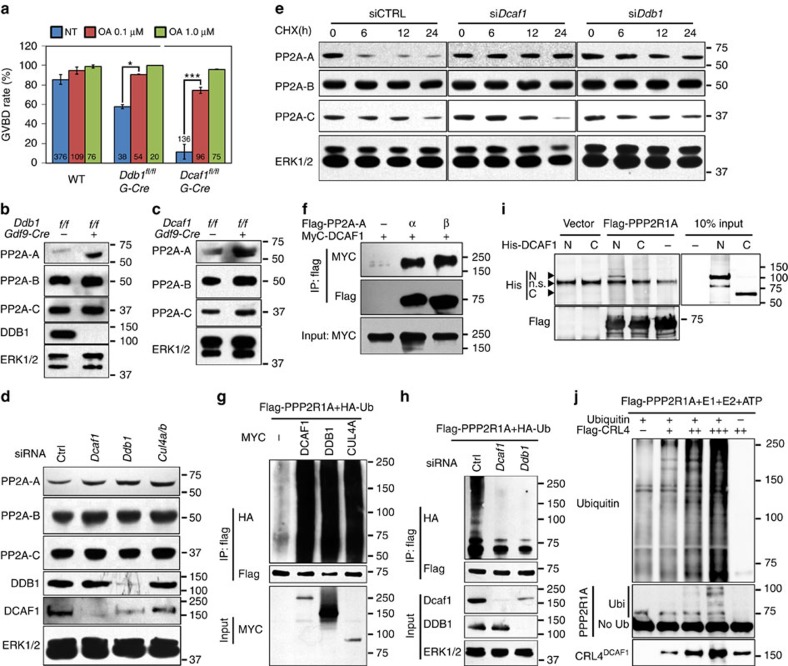
CRL4^DCAF1^ binds to and poly-ubiquinates PP2A-A for degradation. (**a**) Inhibition of PP2A activity by okadaic acid rescued GVBD defects of both *Ddb1*^*oo*−/−^ and *Dcaf1*^*oo*−/−^ oocytes. Total numbers of oocytes used (*n*) are indicated. Error bars indicate s.e.m. *P<0.05, ***P<0.001, Student's *t*-test (**b**,**c**). Western blotting results showing PP2A-A accumulation, but not PP2A-B and -C subunits, in *Ddb1*^*oo*−/−^ (**b**) or *Dcaf1*^*oo*−/−^ (**c**) oocytes at the GV stage. ERK1/2 was used as a protein loading control. (**d**) RNAi depletion of *Dcaf1*, *Ddb1* and *Cul4a/b* in HeLa cells resulted in increased PP2A-A protein levels. Samples were collected at 24 h after siRNA transfection. (**e**) RNAi depletion of *Dcaf1* and *Ddb1* in HeLa cells blocked PP2A-A degradation after cyclohexamide (100 μg ml^−1^) inhibition. Samples were collected at 0, 6, 12 and 24 h after cyclohexamide treatment. (**f**) Co-IP results showing the interaction between DCAF1 and PP2A-A isoforms. HeLa cells were co-transfected with MYC-DCAF1 and FLAG–PPP2R1A/B expression plasmids for 48 h. Target proteins were immunoprecipitated using anti-FLAG agarose beads and subjected to western blotting. Input cell lysates (10%) were immunoblotted with an anti-MYC antibody to show equivalent expression of DCAF1 in all samples. (**g**) PP2A-A poly-ubiquitination levels were increased after overexpressing CRL4^DCAF1^ components. HeLa cells were co-transfected with FLAG–PPP2R1A/B, HA-Ub, and the indicated MYC-tagged protein expression plasmids for 48 h. Target proteins were immunoprecipitated using anti-FLAG agarose beads and subjected to western blotting. (**h**) RNAi depletion of *Dcaf1*/*Ddb1* resulted in reduced PP2A-A poly-ubiquitination. (**i**) *In vitro* binding assay showing that PP2A-A directly interacts with the N terminus of DCAF1. HeLa cells were transfected with FLAG–PPP2R1A expression plasmids for 48 h. Target proteins were immunoprecipitated using anti-FLAG agarose beads. His-tagged N or C terminus of DCAF1 was expressed and purified from *E. coli* cells. n.s., a non-specific band blotted using an anti-His6 antibody, used as a loading control. (**j**) PP2A-A is directly poly-ubiquitinated by CRL4^DCAF1^ *in vitro*. HeLa cells were transfected with FLAG–PPP2R1A or FLAG-DCAF1 expression plasmids for 48 h. Target proteins were immunoprecipitated using anti-FLAG agarose beads separately and subjected to ubiquitination assay in the presence of E1, E2 and Ubiquitin.

In addition to regulating chromosome separation, PP2A is also a repressor of oocyte meiotic resumption^10^,^27^. Interestingly, inhibiting PP2A activity with okadaic acid rescued GVBD in both DDB1- and DCAF1-deleted oocytes (Fig. 3a), which suggested that PP2A was a possible substrate of CRL4^DCAF1^ complexes during meiosis progression.

### CRL4^DCAF1^ induces proteasome degradation of PP2A-A

We next investigated the biochemical effects of CRL4^DCAF1^ activity on PP2A-A degradation. The protein levels of the A subunit (PP2A-A), but not those of the B and C subunits (PP2A-B or PP2A-C), were significantly increased after DDB1/DCAF1 deletion in GV oocytes (Fig. 3b,c and Supplementary Fig. 6a,b). RNA interference (RNAi)-mediated depletion of CRL4 components (*Dcaf1*, *Ddb1* and *Cul4a/b*; Fig. 3d and Supplementary Fig. 6c) increased the protein levels of PP2A-A in HeLa cells. Furthermore, we inhibited *de novo* protein synthesis using cyclohexamide, and then determined the protein degradation rates of PP2A subunits with or without CRL4 activity. PP2A-A was degraded within 12 h after cyclohexamide inhibition in HeLa cells that were transfected with control siRNA, but was stabilized after depleting CRL4 components (Fig. 3e and Supplementary Figs 6d,7a). However, PP2A-B protein levels remained stable throughout 24 h of culture and PP2A-C degradation was not blocked by CRL4 inhibition. Moreover, PP2A-A accumulation was also found in DDB1-deleted hepatocytes collected *in vivo* (Supplementary Fig. 7b). These results indicated that PP2A-A, but not PP2A-B or C was degraded in a CRL4^DCAF1^-dependent manner.

CRL4^DCAF1^ binds to its substrates via the adaptor protein DCAF1. PP2A-A has two isoforms in mammals, PPP2R1A and PPP2R1B. Co-IP results showed that DCAF1 interacted with both PPP2R1A and PPP2R1B (Fig. 3f). Because previous studies reported that PPP2R1A played a more important role than did PPP2R1B in oocytes^10^, we conducted the following experiments primarily using PPP2R1A.

DCAF1 interacts with DDB1 through its WD40 domain, which is close to its C terminus, and binds to its substrate with its long N-terminal domain^15^. Thus, we examined PPP2R1A binding with different DCAF1 fragments. As shown in Supplementary Fig. 7c, DCAF1 bound to PPP2R1A through its N-terminal domains. Interestingly, a migration shift of DCAF1-interacting PPP2R1A was detected. This shifted PPP2R1A band was undetectable in DCAF1-N-fragment pull-down samples (Supplementary Fig. 7c). By western blot analysis using an anti-Ub antibody, we established that this shifted band was ubiquitinated PPP2R1A (Supplementary Fig. 7d).

Overexpressing CRL4 components, DDB1, DCAF1 or CUL4A, greatly increased the poly-ubiquitination status of PPP2R1A (Fig. 3g). By comparison, inhibiting CRL4 activity with MLN4924 (Supplementary Fig. 7e) or siRNA (Fig. 3h) reduced PPP2R1A poly-ubiquitination levels.

Furthermore, we purified FLAG-tagged PPP2R1A in HEK293 cells and His-tagged DCAF1 N-terminal and C-terminal truncated proteins in *Escherichia coli* and subjected to *in vitro* binding assay. PPP2R1A interacted with the N-terminal but not the C-terminal domains of DCAF1 (Fig. 3i). We also did an *in vitro* ubiquitination assay to show that PPP2R1A was directly poly-ubiquitinated by CRL4^DCAF1^. FLAG–PPP2R1A or CRL4^DCAF1^ complex were purified from HEK293 cells separately. FLAG–PPP2R1A was subjected to ubiquitination assay in the presence of CRL4^DCAF1^ or not in the ubiquitination buffer containing recombinant E1, E2, Ub and ATP. As shown in Fig. 3j, PPP2R1A was directly poly-ubiquitinated by CRL4^DCAF1^ in a dose-dependent manner.

These results demonstrated that CRL4^DCAF1^ bound to PP2A-A and resulted in its proteasome degradation due to its poly-ubiquitination. Inhibiting CRL4 activity resulted in PP2A stabilization in both oocytes and somatic cells.

### PP2A-A overexpression mimics DDB1/DCAF1 deletion

**Figure 4:**
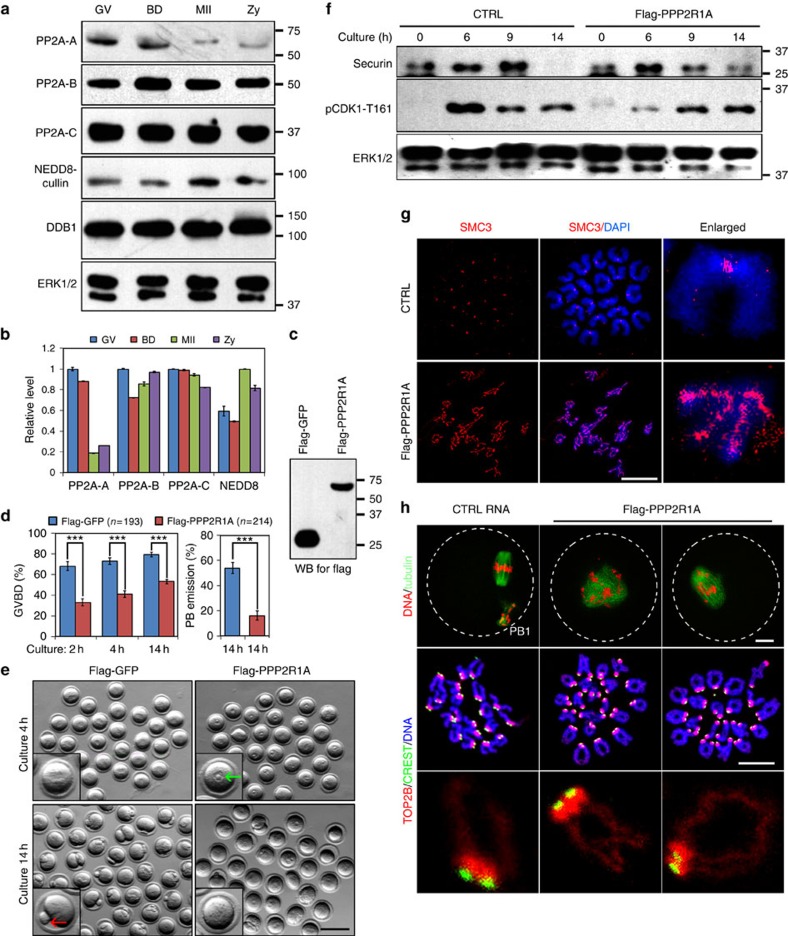
PPP2R1A overexpression in oocytes causes meiotic maturation defects similar to CRL4^DCAF1^ deletion. (**a**,**b**) Western blot results showing levels of the indicated proteins in oocytes at GV, Pro-MI and MII stages, and in zygotes. The band intensities of the indicated proteins were quantified using Image-J software and are shown in (**b**). (**c**) Western blot results showing protein expression after injecting specific mRNAs. Total proteins from 100 injected oocytes were loaded in each lane. (**d**) PP2A-A overexpression in oocytes resulted in reduced GVBD and PBE rates. Numbers of injected oocytes for each mRNA are indicated (*n*). Error bars indicate s.e.m. ***P<0.001, Student's *t*-test. (**e**) Images of oocytes injected with mRNA encoding for FLAG–GFP and FLAG–PPP2R1A at 4 and 14 h after release from GV arrest. Scale bar, 100 μm. Single representative oocytes are shown in the inserts. Green and red arrows indicate GV and PB1, respectively. (**f**) Western blot results showing degradation of securin in PPP2R1A overexpressing oocytes. Oocytes were cultured for the indicated times, and total proteins from 200 oocytes were loaded in each lane. (**g**) SMC3 immunofluorescence (red) on chromosomes of control and PPP2R1A overexpressing oocytes cultured for 14 h. Scale bar, 10μm. (**h**) Microscopic imaging of spindles and chromosomes of oocytes injected with the indicated mRNAs at 14 h after release from GV arrest. Scale bar, 10 μm.

The results described above suggested that CRL4^DCAF1^ might target PP2A-A for degradation and promote meiotic cell cycle progression in oocytes. Among the three PP2A subunits, PP2A-A protein levels were remarkably reduced during oocyte maturation and fertilization, whereas PP2A-B and -C subunits' expression was stable. Interestingly, this change was accompanied by increased CUL4 neddylation or activity (Fig. 4a,b).

To investigate if PP2A-A accumulation was the major factor that contributed to the meiotic maturation defects observed in DDB1- and DCAF1-deleted oocytes, we investigated the effects of PPP2R1A overexpression on mouse oocyte maturation. mRNAs encoding for FLAG–PPP2R1A or FLAG–GFP as a control were injected into WT oocytes that were arrested at the GV stage using milrinone. Their protein expression in oocytes was established by western blot analysis (Fig. 4c). After their release from milrinone, FLAG–PPP2R1A injected oocytes had reduced GVBD and PBE rates (Fig. 4d,e). Consistent with the observations for DDB1-deleted oocytes (Fig. 2b), securin degradation was not blocked, but cohesin removal was impaired in FLAG–PPP2R1A overexpressing oocytes (Fig. 4f,g). For those PPP2R1A overexpressing oocytes that underwent GVBD within 4 h after their release from milrinone, their spindles were disorganized and chromosomes failed to properly align on the equatorial plates (Fig. 4h). Collectively, PPP2R1A overexpression in oocytes mimicked the phenotypes of DDB1- and DCAF1-deleted oocytes.

### PPP2R1A deletion partially rescues DDB1 deletion phenotypes

**Figure 5:**
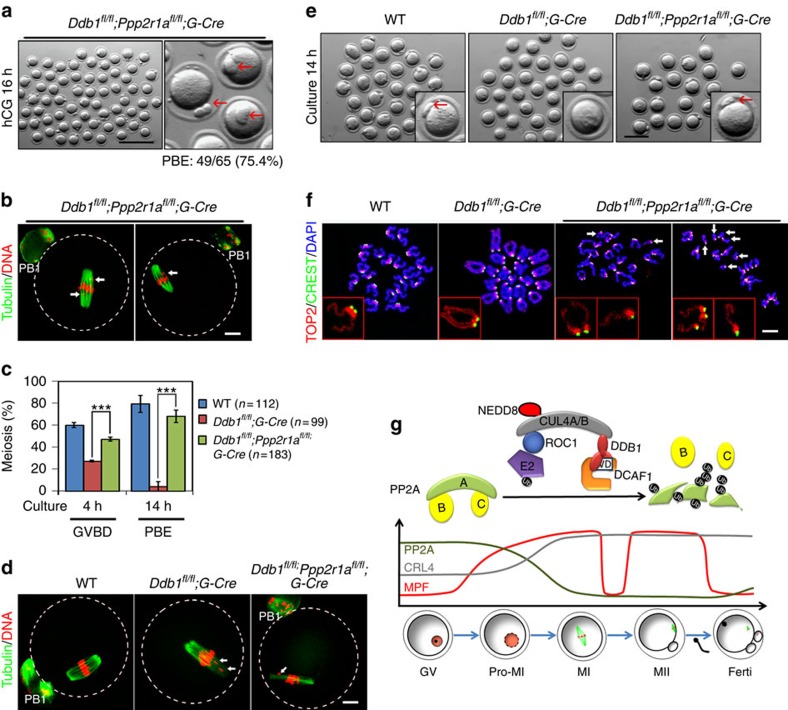
Oocyte-specific *Ppp2r1a* knockout rescues the meiotic defects caused by DDB1 deletion. (**a**) Ovulated oocytes from *Ddb1*/*Ppp2r1a*^*oo*−/−^ females compared with WT and *Ddb1*^*oo*−/−^ oocytes in Fig. 1c. PBE is indicated (75.4%). Red arrows indicate PB1. Scale bar, 200 μm. (**b**) Confocal microscopic images of ovulated *Ddb1*/*Ppp2r1a*^*oo*−/−^ oocytes compared with WT and *Ddb1*^*oo*−/−^ oocytes in Fig. 1e. Oocyte outlines are highlighted by broken lines. Arrows indicate misaligned chromosomes. Scale bar, 10 μm. (**c**) *In vitro* GVBD and PBE rates of GV oocytes collected from mice with the indicated genotypes. PBE was analysed only for oocytes that underwent GVBD within 4 h. Error bars indicate s.e.m. and the numbers of analysed oocytes are indicated. ***P<0.001, Student's *t*-test. (**d**) Confocal microscopic images of WT, *Ddb1*^*oo*−/−^ and *Ddb1*/*Ppp2r1a*^*oo*−/−^ oocytes after *in vitro* culture for 14 h. Scale bar, 10 μm. (**e**) Morphologies of oocytes from mice with the indicated genotypes after *in vitro* culture for 14 h. Single representative oocytes are shown in the inserts. Red arrows indicate PB1.Scale bar, 100 μm. (**f**) Representative chromosome configurations of the indicated genotypes. GV oocytes were cultured for 14 h and then used to prepare chromosome spreads. Precociously segregated sister chromatids are indicated by arrows. Enlarged chromosomes are shown in the inserts. Scale bar, 5 μm. (**g**) Schematic showing CRL4^DCAF1^ function for regulating PP2A-A degradation to facilitate oocyte meiotic progression.

To confirm that PPP2R1A was a physiological CRL4 substrate, we generated oocyte-specific *Ppp2r1a* and *Ddb1* double knockout mice (*Ddb1*^*fl/fl*^*;Ppp2r1a*^*fl/fl*^*;Gdf9-Cre*, referred to as *Ddb1*/*Ppp2r1a*^*oo*−/−^). Interestingly, the oocytes ovulated by *Ddb1*^*oo*−/−^ mice did not have a PB1 (Fig. 1c), whereas 75.4% of oocytes ovulated by *Ddb1*/*Ppp2r1a*^*oo*−/−^ females had a PB1 (Fig. 5a). Immunofluorescent staining for α-tubulin and DNA showed that PB1 was extruded and normally shaped MII spindles formed in *Ddb1*/*Ppp2r1a*^*oo*−/−^ oocytes (Fig. 5b). The WT control results were similar to those shown in Fig. 1c,e. However, some chromosomes failed to align at the equatorial plate (Fig. 5b, indicated by arrows), as previously reported for *Ppp2r1a*^*oo*−/−^ oocytes^10^.

When cultured *in vitro*, *Ddb1*/*Ppp2r1a*^*oo*−/−^ oocytes had a remarkably higher GVBD rate (61.8%) than did in *Ddb1*^*oo*−/−^ oocytes (38.7%). However, their GVBD rate was still lower than that of WT oocytes (80.3%; Fig. 5c). Cultured *Ddb1*/*Ppp2r1a*^*oo*−/−^ oocytes had a PB1 emission rate (68%) comparable to that of WT controls (79%). This was in sharp contrast to that of *Ddb1*^*oo*−/−^ oocytes (1.1%; Fig. 5c–e). In addition, homologous chromosomes were separated in *Ddb1*/*Ppp2r1a*^*oo*−/−^ oocytes (Fig. 5f). Moreover, precocious separation of sister chromatids was also observed in these oocytes. The chromosomes of *Ddb1*/*Ppp2r1a*^*oo*−/−^ oocytes were a mixture of tetrads (5.1%), dyads (85.3%) and single chromatids (9.6%; Fig. 5f). This phenotype was previously reported for *Ppp2r1a*^*oo*−/−^ oocytes. Collectively, these results indicated that PPP2R1A was a physiological CRL4^DCAF1^ substrate in oocytes and that its abnormal accumulation contributed to most of the meiotic maturation-related defects that were observed in CRL4^DCAF1^-deleted oocytes.

## Discussion

We previously reported that oocyte-specific deletion of *Ddb1* or its substrate adaptor *Dcaf1* resulted in a rapid loss of ovarian oocyte reserves and premature ovarian insufficiency at young adulthood^19^,^20^. CRL4^DCAF1^ activated ten-eleven translocation (TET) DNA hydroxymethylases to regulate DNA methylation and the expression of oocyte-specific genes^19^,^20^. However, not all *Ddb1*^*oo*−/−^ mouse phenotypes could be explained by this mechanism. *Ddb1*^*fl/fl*^*;Gdf9-Cre* females were infertile even before their oocytes were exhausted, which was evident at about 3 months after birth. Embryos derived from DDB1-deleted oocytes failed to develop beyond the four-cell stage^19^. This defect was more severe than maternal deletion of TET3 (ref. 28). Thus, mechanisms other than TET inactivation must have contributed to the infertile phenotype of *Ddb1*^*oo*−/−^ female mice.

In this study we found that CRL4^DCAF1^ controlled oocyte meiotic progression as early as 3 weeks after birth, before genomic DNA hypomethylation-caused oocyte gene silencing. These effects were primarily caused by PP2A-A accumulation, as *Ppp2r1a* knockout in oocytes rescued most of the meiosis defects resulting from DDB1 deletion (summarized in Supplementary Fig. 8). PP2A acted as a guardian of GV arrest and sister chromatid cohesin during meiosis I (refs 10, 29). However, the regulation of PP2A activity during meiotic progression remained elusive. PP2A reportedly interacted with a proteasome Ub system in fission yeast^30^. Mass spectroscopy studies identified an interaction between a PP2A-Aα subunit (PPP2R1A) and CRL4 E3 Ub ligase^18^. On the basis of these clues, we identified a novel mechanism by which PP2A was regulated by CRL4^DCAF1^-mediated proteasome degradation during oocyte meiotic maturation. This is the first study to show a function for CRL4 *in vivo* by regulating chromosome separation and cell division. Our oocyte-specific gene knockout mouse models and PP2A-A overexpression results indicated that CRL4-mediated PP2A degradation was physiologically important for controlling prophase I arrest and prometaphase progression during meiosis.

We also observed that PP2A accumulation in oocytes inhibited SAC activation. During Pro-MI, SAC is activated to inhibit the premature activation of APC/C complexes before successful chromosome alignment. However, in DDB1-deleted oocytes, SAC was not activated when cultured for 8 h and APC complex was activated. This was consistent with the results of previous reports that PP2A directly or indirectly inhibited the phosphorylation of SAC components, thereby causing SAC inactivation^11^,^31^. In CRL4^DCAF1^-deleted oocytes, homologous chromosomes were misaligned on the spindles and microtubules failed to pull them apart. Securin was also degraded, which suggested separase activation. Owing to the ectopic expression of PP2A on chromosome arms, separase failed to separate homologous chromosomes during anaphase I.

A recent study investigated the physiological role of microtubule associated serine/threonine kinase-like (MASTL or greatwall kinase), a PP2A suppressor, in mouse oocytes^32^. It was found that MASTL was required for the timely activation of APC during meiosis I exit and was indispensable for meiotic resumption. However, in *Mastl*^*oo*−/−^ oocytes, PP2A activity was not dramatically increased as compared with that in WT oocytes at the GV stage and after GVBD. In these oocytes, PP2A activity was only significantly increased after PBE^32^. These results suggested that MASTL primarily inhibited PP2A activity at the relatively later stages of oocyte maturation (metaphase I–II transition). Consistent with these results, delayed PBE was also reported for *Mastl*^*oo*−/−^ oocytes. We observed a related phenotype in that homologous chromosome separation and PBE were blocked in *Ddb1*^*oo*−/−^ oocytes or PP2A overexpressing oocytes. These two independent studies suggested that excessive PP2A activation caused these oocyte maturation defects.

A study by Liu *et al*. and our results suggested that PP2A activity might be controlled by two distinct mechanisms in mouse oocytes. One is a post-translational modification by which MASTL phosphorylates and stabilizes the PP2A inhibitors endosulfine alpha and cAMP-regulated phosphoprotein 19. After phosphorylation, these inhibit PP2A activity to promote anaphase^33^. The other is CRL4-mediated degradation of the PP2A scaffold subunit, which reduces PP2A activity to facilitate non-reversible meiotic progression. These two regulation mechanisms of PP2A activity in conjunction with other meiotic regulators ensure precise meiotic progression in oocytes.

This is the first study to report on the functions of CRL4 during metaphase/anaphase of the cell cycle. By forming more than 90 distinct E3 Ub ligases with different substrate adaptors, CRL4 targets different substrates for degradation^16^. Among these, DNA replication licensing factor CDT1 and cyclin-dependent kinase inhibitor 1 (p21) are widely regarded as cell cycle regulators^34^. In somatic cells, ablating CRL4 components causes DNA damage, which arrests the cell cycle before entry into metaphase^35^,^36^. These CRL4 functions during interphase restricted investigating its function during metaphase and identifying its new substrates during the cell cycle.

Oogenesis is a specialized cell cycle that involves a long division process (>12 h, and ∼30 min in mitosis). Oocytes enter meiosis and are arrested at the diplotene stage of prophase I early during foetal life. Thus, by using *Gdf9-Cre* transgenic mice^37^, we could delete DDB1 and DCAF1 in oocytes at the GV stage without causing genomic DNA damage. In this way, we could investigate the specific roles of CRL4 in dividing cells.

PP2A-A protein levels are reduced after GVBD. Thus, we hypothesized that CRL4 activity should be regulated during meiosis. However, it was difficult to determine the CRL4 Ub ligase activity at different stages because the small numbers and sizes of mouse oocytes prevented the purification of endogenous CRL4. The cullin family E3 Ub ligases require a NEDD8 modification to acquire their Ub ligase activity. CRLs activity are controlled by cycles of neddylation, CAND1 binding, and the abundance of adaptor modules^18^,^38^. Interestingly, CUL4 neddylation was increased after meiotic resumption. Therefore, on meiotic resumption, CRL4 activity might be upregulated to target PP2A for degradation and facilitate meiotic progression.

Our findings with oocytes have potentially important implications for somatic cells. In HeLa cells, PP2A-A was also ubiquitinated and degraded in a CRL4^DCAF1^-dependent manner. These results indicated that CRL4 might also control the mitotic cell cycle by inhibiting PP2A activity. PP2A is considered to be a tumour suppressor and has important roles during tumorigenesis. PPP2R1A and PPP2R1B are mutated in many cancers, including breast, colon and lung carcinomas and melanoma^39^,^40^. Reduced PP2A activity facilitates the ectopic activation of oncogenes, including RalA, β-catenin, BCL2, c-Myc and others^41^,^42^,^43^. CRL4 components were found to be highly expressed in many types of cancer cells and were required for their proliferation^35^. By identifying PP2A as a novel CRL4 target, our study results provide new insights into the roles of CRL4 during tumorigenesis.

## Methods

### Mice

WT C57/B6 mice were from the Zhejiang Academy of Medical Science, China. *Ddb1*^*flox/flox*^*;Gdf9-Cre* and *Dcaf1*^*flox/flox*^*;Gdf9-Cre* mice were generated by crossing *Ddb1* and *Dcaf1* floxed mice to *Gdf9-Cre* transgenic mice. Genotyping primers for *Ddb1*^*flox*^ alleles were 5′-CGGGACTGGAGCATTTTTGACTAC-3′ and 5′-ATTTTCTGTGTATGGAGGGGAGTG-3′ (388 bp for WT allele and 430 bp for floxed allele) and primers for *Dcaf1*^*flox*^ alleles were 5′-CAGTTAGAGAGTGACTTTGGACG-3′ and 5′-GCTGCCAACTATGGGTGC-3′ (433 bp for WT allele and 470 bp for floxed allele). Primers 5′-GGTTTCTGTTGGGCTCTCAC-3′ and 5′-ATCAGAGGTGGCATCCACAG-3′ were used to produce a 470 bp band to identify *Gdf9-Cre*. *Ppp2r1a*^*flox/flox*^ mice were purchased from the Jackson Laboratory and genotyped by using primers 5′-AGGACAAGTCTTGGCGTG-3′ and 5′-GAATTAAACCCAGGACCCCTGG-3′ (671 bp for WT allele and 825 bp for floxed allele). Mice were maintained under specific pathogen free conditions in a controlled environment of 20–22 °C, with a 12/12 h light/dark cycle, 50–70% humidity, and food and water provided *ad libitum*. Animal care and experimental procedures were conducted in accordance with the Animal Research Committee guidelines of Zhejiang University. *Ddb1*-deleted liver tissues were obtained from *Ddb1*^*fl/fl*^*;Mx1-Cre* mice 2 weeks after Poly(I:C) injection^44^. All mutant mouse strains had a C57BL/6 background.

### Antibodies

Anti-DDB1, Bethyl (IHC-0013401), IHC (1:400), IF (1:200); Anti-DDB1, Epitomics (3821-1), WB (1:10,000); Anti-DCAF1, Proteintech (11612-1-AP), IHC (1:200), IF (1:200), WB (1:1,000); Anti-MYC, Cell Signaling (2272), WB (1:1,000); Anti-HA, Cell Signaling (3724), WB (1:1,000); Anti-HA, Sigma (F3165), WB (1:1,000); Anti-ERK1/2, Santa Cruz (sc-94), WB (1:1,000); anti-FITC-α-tubulin, Sigma (F2168), IF(1:500); anti-CREST, Fitzgerald Industries International (90C-CS1058), IF (1:100); anti-TOP2B, Epitomics (3747-1), IF (1:200); anti-SMC3, Abcam (ab128919), IF (1:20); anti-MAD2, Abcam (ab9777), IF (1:100); anti-PP2A-A, Cell Signaling (2041), WB (1:1,000), IF (1:100); anti-PP2A-B, Cell Signaling (2290), WB (1:1,000); anti-PP2A-C, Cell Signaling (2259), WB (1:1,000); anti-NEDD8, Epitomics (1571-1), WB (1:1,000); anti-cyclin B, Cell Signaling (4138), WB (1:1,000); anti-securin, Abcam (ab3305), WB (1:1000); anti-CDC20, Santa Cruz (sc-8358), WB (1:500); anti-pCDK1(T161), Cell Signaling (9114), WB (1:1,000); anti-pERK1/2, Cell Signaling (9101), WB (1:1,000).

### Histological analysis and Immunohistochemistry

Mouse ovaries were fixed overnight in 10% PBS-buffered formalin, dehydrated and embedded in paraffin. Ovary samples were serially sectioned (5 μm thick) and stained with hematoxylin and eosin. GVBD rates were determined only for large preovulatory follicles that contained oocytes with clearly visible nuclei at 4 h after hCG injection.

For immunohistochemistry, sections were deparaffinized, rehydrated, and incubated with primary antibodies at room temperature for 1 h, then reacted with biotin-labelled secondary antibodies for 30 min, and finally stained using Vectastain ABC kits and DAB peroxidase substrate kits (Vector Laboratories, Burlingame, CA).

### Superovulation and fertilization

For superovulation, 21–23-day-old female mice were injected intraperitoneal with 5 international units (IU) of PMSG (Ningbo Sansheng Pharmaceutical, China) and 44 h later with 5 IU of hCG (Ningbo Sansheng Pharmaceutical, China). After an additional 16 h, oocyte/cumulus masses were surgically removed from oviducts and the numbers of oocytes were counted after digestion with 0.3% hyaluronidase (Sigma-Aldrich). Oocyte morphology was observed and images were acquired with a Nikon SMZ1500 stereoscope.

To obtain early embryos, female mice were mated with 10–12-week-old WT males. Successful mating was confirmed by the presence of vaginal plugs. Zygotes or two-cell embryos were collected from oviducts at the indicated times post-hCG injection.

### Confocal microscopy for mouse oocytes

Oocytes were fixed in PBS-buffered 4% paraformaldehyde for 30 min at room temperature, followed by permeabilization with 0.2% Triton X-100. After blocking with 1% bovine serum albumin in PBS, oocytes were incubated with primary antibodies diluted in blocking solution at room temperature for 1 h. After three washes with PBS, oocytes were labelled with secondary antibodies for 45 min, and then counterstained with 5 μg ml^−1^ of 4′,6-diamidino-2-phenylindole (DAPI) or propidium iodide (Molecular Probes, Life Technologies, Carlsbad, CA, USA) for 10 min. Oocytes were mounted on glass slides using SlowFade Gold Antifade Reagent (Life Technologies) and examined with a confocal laser scanning microscope (Zeiss LSM 710, Carl Zeiss AG, Germany).

### Preparation of chromosome spread sections

Oocytes at different meiosis stages were collected and the zona pellucida was removed using acidic M2 medium (pH 2.0). Then, oocytes were fixed in a CS solution that contained 1% paraformaldehyde, 0.15% Triton X-100 and 3 mM dithiothreitol on glass slides for 30 min. Successful oocyte rupture was monitored under a stereoscope. After fixation, slides were air dried. Immunofluorescent staining of chromosome and centromere proteins was performed as done for oocytes as described above.

### Oocyte culture, microinjection and live imaging

Mice at 21-days of age were injected with 5 IU of PMSG and humanely euthanized 44 h later. Oocytes at the GV stage were collected in M2 medium (M7167; Sigma-Aldrich) and cultured in mini-drops of M16 medium (M7292; Sigma-Aldrich) covered with mineral oil (M5310; Sigma-Aldrich) at 37 °C in a 5% CO_2_ atmosphere. For microinjection, oocytes were collected in M2 medium with 2.5 μM milrinone to inhibit spontaneous GVBD. mRNAs were transcribed *in vitro* using a SP6 message machine kit (Invitrogen, AM1450). Microinjection was performed using an Eppendorf microinjector.

For live imaging, mRNAs encoding for RFP-H2B, GFP-tubulin and mCherry-securin were microinjected into WT and DDB1-deleted oocytes and released from milrinone after 2 h. Images of live oocytes were acquired on a DV ELITE High Resolution Invented Living Cell Workstation and imaged at 5 min intervals for 16 h.

### Western blot analysis

Protein samples were separated by SDS-PAGE and electrophoretically transferred to PVDF membranes (Millipore). After incubation with primary antibodies followed by an HRP-linked secondary antibody, bands on membranes were disclosed using an Enhanced Chemiluminescence Detection Kit (Amersham). Full-sized uncropped blots of cropped blots used in figures are included in Supplementary Fig. 9.

### Cell culture, plasmid transfection and RNA interference

HeLa cells and HEK293 cells were grown in DMEM (Invitrogen) supplemented with 10% foetal bovine serum (Hyclone) and 1% penicillin-streptomycin solution (Gibco) at 37 °C in a humidified 5% CO_2_ incubator. The neddylation inhibitor MLN4924 (kind gift of Dr L.J. Jia) was added to cell culture medium at concentrations of 0.1–1 μM. Transient plasmid transfection was done using Lipofectamine 2000 (Invitrogen). RNAi experiments were done using siRNA duplexes (Microsynth, Balgach, Switzerland) to deplete endogenous *Dcaf1*, *Ddb1*, or *Cul4a/b* in HeLa cells. siRNA sequences are 5′-UCACAGAGUAUCUUAGAGATT-3′ for *Dcaf1*, 5′-GGCCAAGAACAUCAGUGUGTT-3′ for *Ddb1*, 5′-GAAGCUGGUCAUCAAGAACdTdT-3′ for *Cul4a* and 5′-AAGCCUAAAUUACCAGAAAdTdT-3′ for *Cul4b*.

### Immunoprecipitation

At 48 h after transfection, cells were lysed in lysis buffer (50 mM Tris-HCl, pH 7.5, 150 mM NaCl, 10% glycerol and 0.5% NP-40; protease and phosphatase inhibitors were added before use). After centrifugation at 12,000*g* for 10 min, the supernatant was subjected to immunoprecipitation with an anti-FLAG M2 affinity gel (Sigma). After incubation at 4 °C for 4 h, beads were washed three times with lysis buffer. SDS sample buffer was added to the beads and the eluates were used for western blot analysis.

### Ubiquitination assay

To detect protein ubiquitination status, cells were lysed in denaturing buffer (20 mM Tris, pH 7.4, 50 mM NaCl, 0.5% NP-40, 0.5% sodium deoxycholate, 0.5% SDS and 1 mM EDTA; protein inhibitors were added before use) followed by sonication. After centrifugation, the supernatant was used for immunoprecipitation.

For an *in vitro* ubiquitination assay, plasmids encoding for either FLAG–PPP2R1A or FLAG-DCAF1 were transfected into HEK293T cells. Cells were lysed in NP-40 buffer. FLAG–PPP2R1A and CRL4^FLAG-DCAF1^ were immunopreciptated with an anti-FLAG M2 affinity gel (Sigma) and eluted with FLAG peptides. Reactions were performed at 37 °C for 1 h in the presence of His-Ub, E1, E2 and ATP. Reactants were diluted with denaturing buffer and re-immunopreciptated with an anti-FLAG M2 affinity gel. The immunoprecipitants were subjected to SDS-PAGE and immunoblotted with Ub and His6-tag antibodies.

### Statistical analysis

Results are given as means±s.e.m.; each experiment included at least three independent samples and was repeated at least three times. Results for two experimental groups were compared by two-tailed unpaired Student's *t*-tests. In results and figures, *P*<0.05, *P*<0.01 and *P*<0.001 are indicated by one asterisk (*), two asterisks (**) and three asterisks (***), respectively.

## Additional information

**How to cite this article**: Yu, C. *et al*. CRL4–DCAF1 ubiquitin E3 ligase directs protein phosphatase 2A degradation to control oocyte meiotic maturation. *Nat. Commun.* 6:8017 doi: 10.1038/ncomms9017 (2015).

## Supplementary Material

Supplementary Figures Supplementary Figure 1-9

Supplementary Movie 1 Live cell imaging results of securin degration in WT oocytes that are injected with mCherry-securin (red) and GFP-tubulin (green).

Supplementary Movie 2 Live cell imaging results of securin degration in DDB1-deleted oocytes that are injected with mCherry-securin (red) and GFP-tubulin (green).
