# Supplementary material for: CRL4–DCAF1 ubiquitin E3 ligase directs protein phosphatase 2A degradation to control oocyte meiotic maturation
Source: Nat Commun. 2015 Aug 18;6:8017. doi: 10.1038/ncomms9017 (PMC4557334; doi:10.1038/ncomms9017)
Supplement: Supplementary Figures — Supplementary Figure 1-9 [file ncomms9017-s1.pdf]

Supplementary information

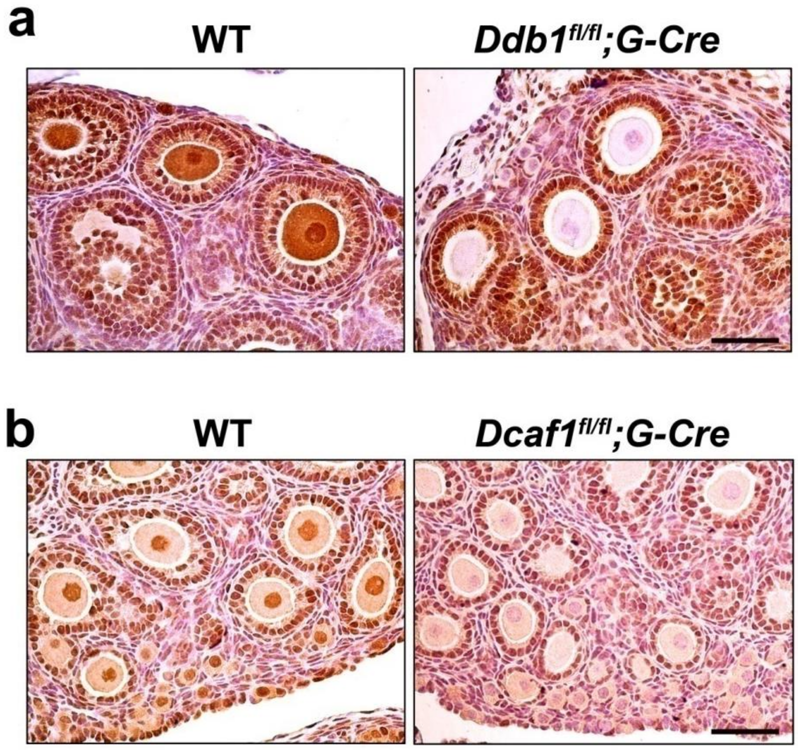

Supplementary Figure 1. Oocyte-specific *Ddb1* and *Dcaf1* knockout.

Immunohistochemistry results showing successful deletion of DDB1 **(a)** and DCAF1 **(b)** in oocytes of *Ddb1*$^{fl/fl}$*;Gdf9-Cre* and *Dcaf1*$^{fl/fl}$*;Gdf9-Cre* ovaries at PD21, respectively. Scale bar, 100 μm.

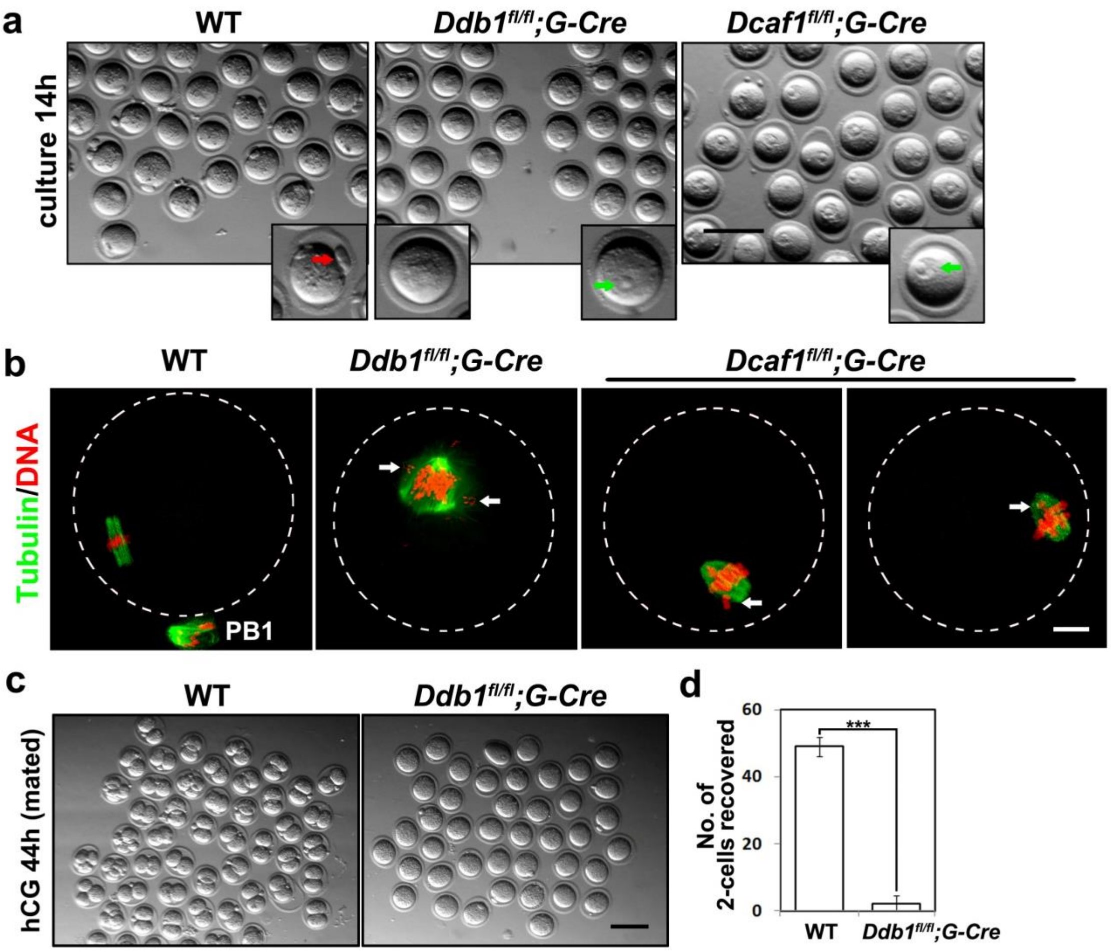

Supplementary Figure 2. Oocyte meiotic maturation defects of *Ddb1$^{fl/fl}$;Gdf9-Cre* and *Dcaf1$^{fl/fl}$;Gdf9-Cre* females. (a) Images of WT (n=207), *Ddb1$^{fl/fl}$;Gdf9-Cre* (n=288) and *Dcaf1$^{fl/fl}$;Gdf9-Cre* (n=94) oocytes after culture for 16 hours *in vitro*. Representative oocytes are enlarged at lower right. GVBD and PBE rates were shown in Fig. 1f. Green and red arrows indicate GV and PB1, respectively. Scale bar, 100 µm. **(b)** α-tubulin (green) and DNA (red) staining showing disrupted spindles and misaligned chromosomes in ovulated oocytes of *Ddb1$^{oo-/-}$* or *Dcaf1$^{oo-/-}$* females. Dashed lines indicate oocyte outlines and arrows indicate lagging chromosomes. PB1, first polar body. Scale bar, 10 µm. **(c)** DDB1 deleted oocytes were not fertilized and did not initiate embryogenesis, whereas WT oocytes developed to the 2-cell embryo stage after fertilization (n=3 for each genotype). Scale bar, 100 µm. **(d)** Numbers of 2-cell embryos recovered from oviducts per mouse at 44 h after hCG injection. For each genotype, 5 mice were analyzed. Error bars indicate S.E.M.'s.

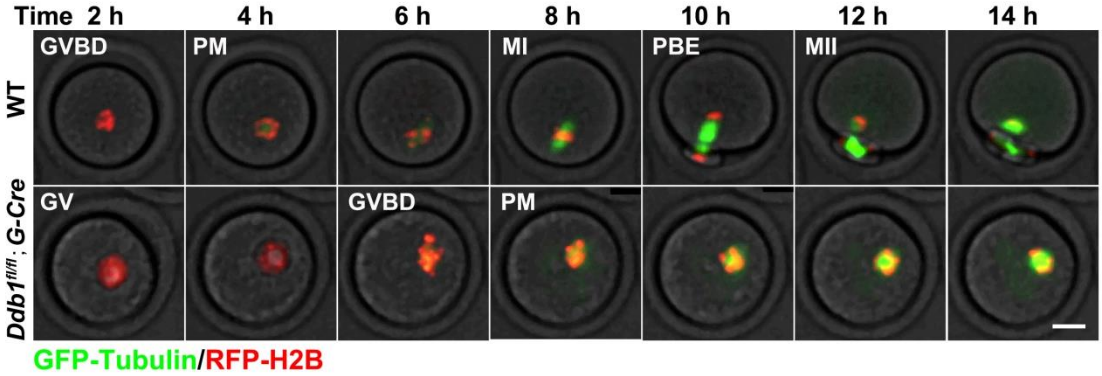

Supplementary Figure 3. Live imaging results showing *in vitro* meiotic division of WT and DDB1-deleted oocytes. Time after release from milrinone is indicated (h). About 40 WT and DDB1-deleted oocytes were subjected to live cell imaging. Abbreviations: GV, germinal vesicle; GVBD, GV breakdown; PM, prometaphase; MI, metaphase I; PBE, polar body extrusion; and MII, metaphase II. Scale bar, 20 µm.

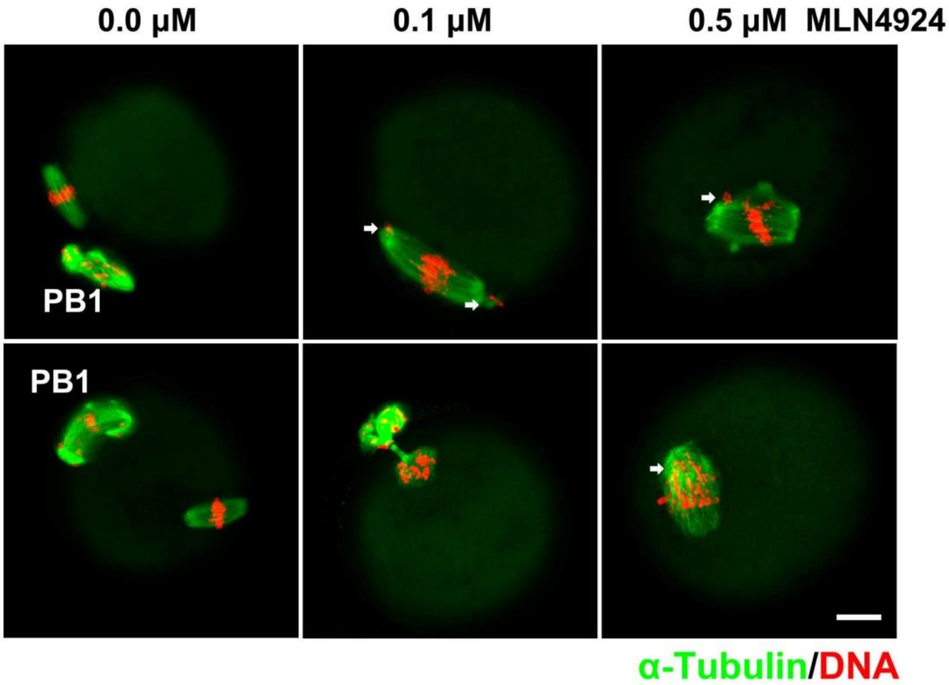

Supplementary Figure 4. Inhibition of CRL activity by MLN4924 disrupts meiotic spindles and polar body extrusion. Arrows indicate lagging chromosomes. PB1, first polar body. Scale bar, 10 µm.

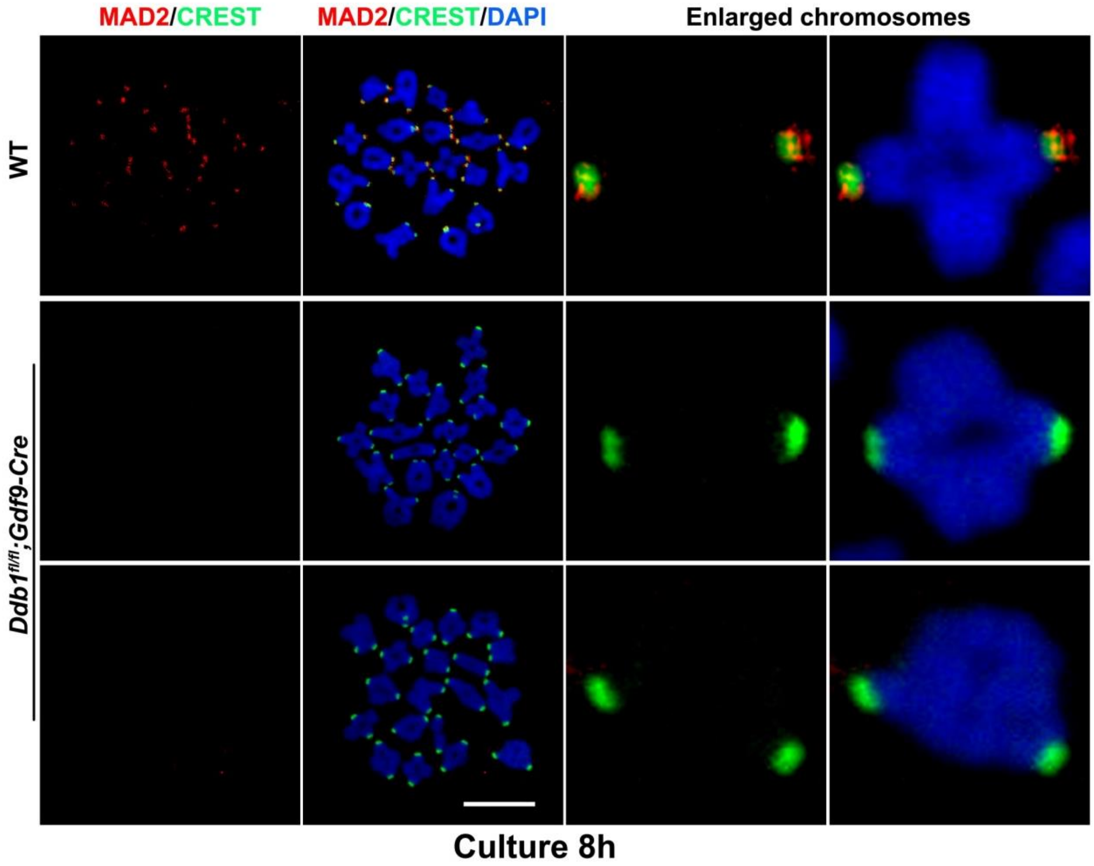

Supplementary Figure 5. Immunofluorescent staining of MAD2 (red) on chromosomes of WT and *Ddb1$^{oo-/-}$* oocytes cultured for 8 hours. MAD2, a core component of spindle assembly checkpoint (SAC), was localized on centromeres in WT oocytes at the Pro-MI stage when not all the chromosome pairs aligned at the equator plates. But in *Ddb1$^{oo-/-}$* oocytes, the localization of MAD2 on centromeres was not detected, which indicates that SAC was not activated in *Ddb1$^{oo-/-}$* oocytes. CREST was immunostained (green) to label centromeres and DNA was stained in blue. Scale bar, 5 μm.

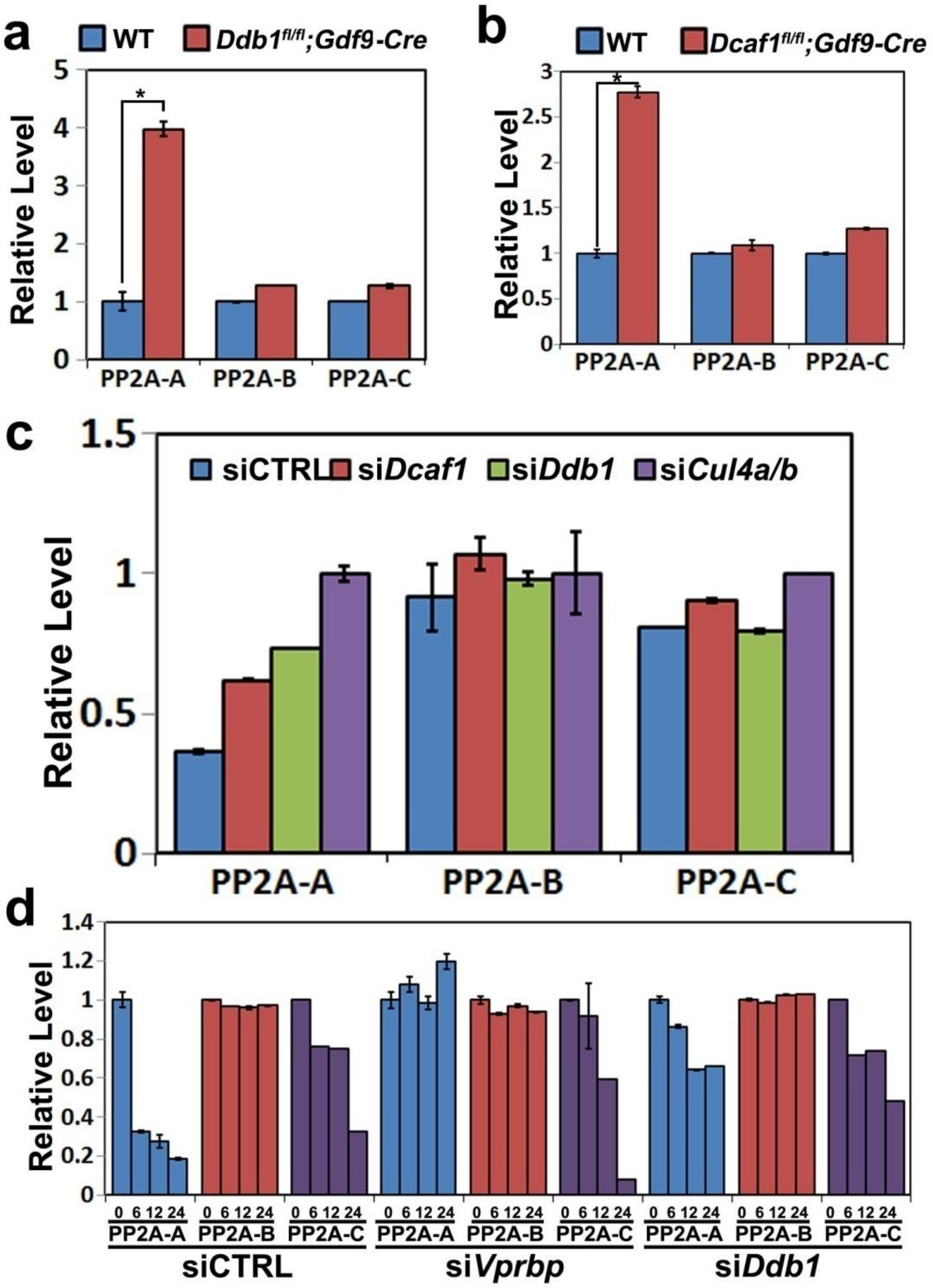

Supplementary Figure 6. Quantitative band intensities of Western blot results. (a, b) Quantitative results for the relative protein levels of PP2A subunits in $\textit{Ddb1}^{oo-/-}$ and $\textit{Dcaf1}^{oo-/-}$ oocytes. The relative intensity of each band shown in (Fig. 3b, c) was quantified using Image J software. **(c, d)** Quantitative results for relative protein levels of PP2A subunits in Fig. 3d **(c)** and Fig. 3e **(d)**.

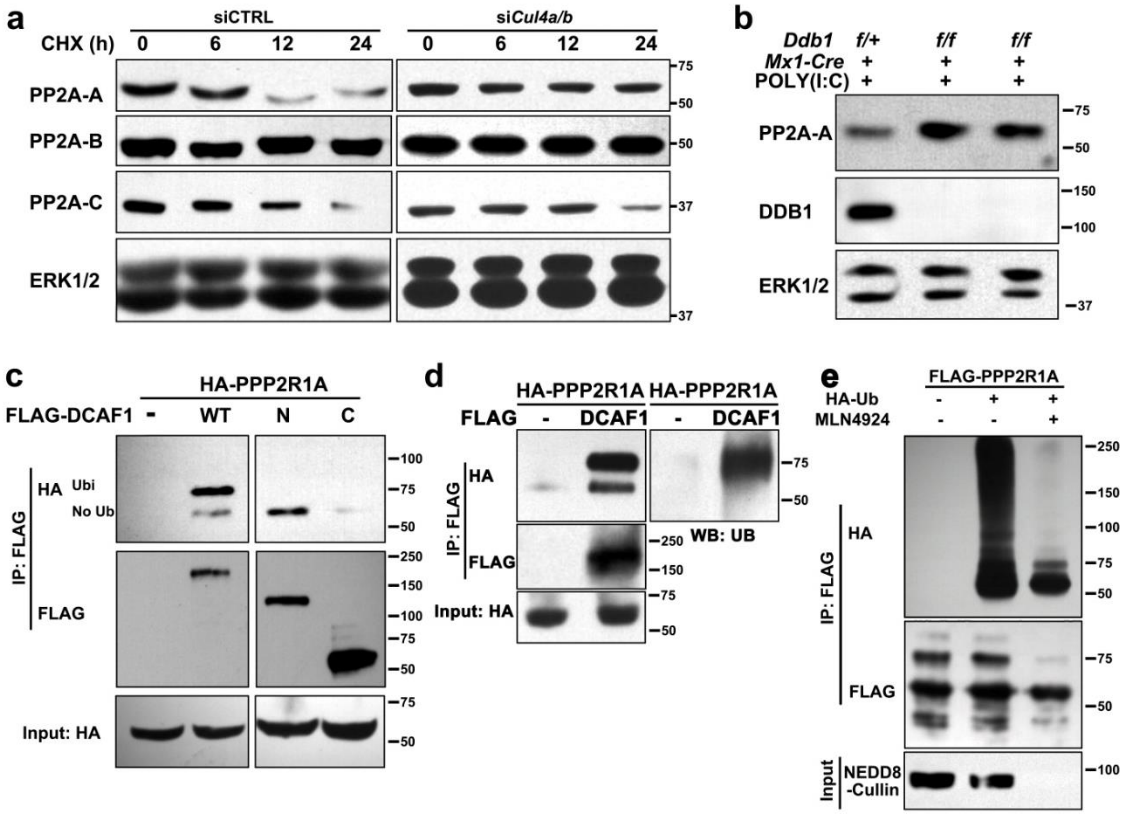

Supplementary Figure 7. Interaction between PP2A-A and DCAF1. **(a)** RNAi depletion of *Cul4a/b* in HeLa cells blocked PP2A-A degradation after cyclohexamide (CHX, 100 µg/ml) inhibition. Samples were collected at 0, 6, 12, and 24 h after CHX treatment. **(b)** Western blot results showing PP2A-A accumulation in DDB1-deleted hepatocytes. **(c)** Co-IP results showing that PP2A-A interacts with the N terminus of DCAF1.HeLa cells were co-transfected with HA-PPP2R1A and FLAG-DCAF1 expression plasmids for 48 h. Target proteins were immunoprecipitated using anti-FLAG agarose beads and subjected to Western blotting. **(d)** Western blot results showing that DCAF1-interacting PPP2R1A is ubiquitinated. **(e)** MLN4924 inhibition reduces PP2A-A poly-ubiquitination.

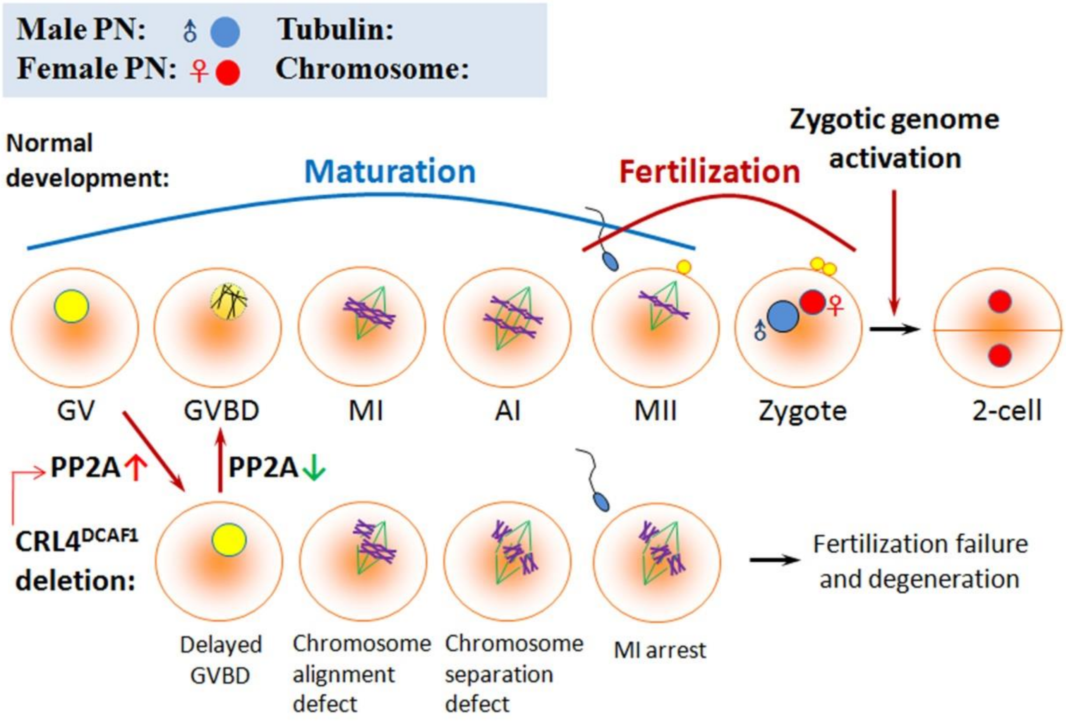

Supplementary Figure 8. Schematic showing CRL4^{DCAF1} function during mouse oocyte meiotic maturation. CRL4^{DCAF1} deletion in mouse oocytes results in PP2A accumulation, which results in delayed GVBD and defects in chromosome alignment and separation. Simultaneously deleting PP2A and CRL4^{DCAF1} activity rescues most phenotypes caused by CRL4^{DCAF1} deletion.

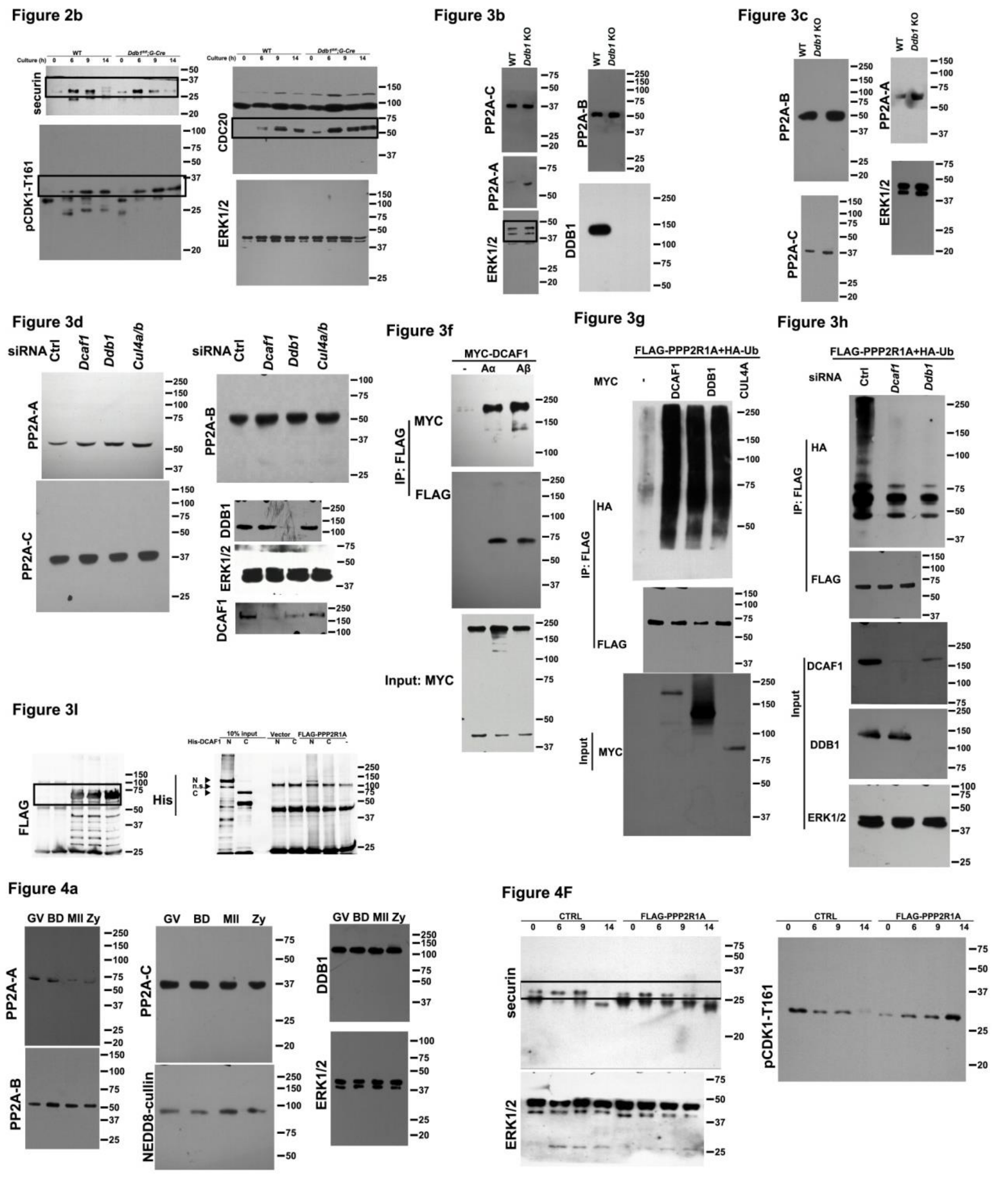

Supplementary Figure 9. Uncropped scans of the most important Western blot results. Black boxes highlight lanes used in figures.
